# A Teleneuropsychology Protocol for the Cognitive Assessment of Older Adults During COVID-19

**DOI:** 10.3389/fpsyg.2021.651136

**Published:** 2021-05-13

**Authors:** Marcela Kitaigorodsky, David Loewenstein, Rosie Curiel Cid, Elizabeth Crocco, Katherine Gorman, Christian González-Jiménez

**Affiliations:** Department of Psychiatry and Behavioral Sciences, Center for Cognitive Neuroscience and Aging, University of Miami, Miami, FL, United States

**Keywords:** teleneuropsychology, telehealth, protocol, COVID-19, older adults, dementia

## Abstract

The Coronavirus Disease 2019 (COVID-19) pandemic prompted the need for a teleneuropsychology protocol for the cognitive assessment of older adults, who are at increased risk for both COVID-19 and dementia. Prior recommendations for teleneuropsychological assessment did not consider many of the unique challenges posed by COVID-19. The field is still in need of clear guidelines and standards of care for the assessment of older adults under the current circumstances. Advantages of teleneuropsychological assessment during the COVID-19 pandemic include reduced risk of contracting the virus, eliminating travel time and reducing cost, and more rapid access to needed services. Challenges include disparities in technology access among patients, reduced control over the testing environment, impeded ability to make behavioral observations, and limited research on valid and reliable cognitive assessment measures. The aim of this perspective review is to propose a teleneuropsychological protocol to facilitate neuropsychological assessment utilizing a virtual platform. The proposed protocol has been successful with our clinical and research populations and may help neuropsychologists implement teleneuropsychology services without compromising validity or reliability. However, there is increasing need for research on teleneuropsychological assessment options for both clinical and research purposes.

## Introduction

The Coronavirus Disease (COVID-19) pandemic has posed major challenges for healthcare systems around the world. According to the Center for Disease Control and Prevention (CDC), older adults are particularly vulnerable to developing severe complications from the virus and have the highest risk of hospitalization and death (Centers for Disease Control and Prevention, 2021). Thus, older adults have been strongly advised to adhere to the most restrictive social distancing guidelines, often limiting their ability or willingness to seek medical services. The abovementioned issues have been exacerbated for those with dementia, who have an even greater risk of morbidity and mortality (Verity et al., 2020; Williamson et al., 2020; Zhou et al., 2020). Alzheimer’s Disease (AD), the most common form of dementia, affects approximately 5.8 million people in the United States (Alzheimer’s Association, 2020). The prodromal stage of AD, coined mild cognitive impairment (MCI), is a symptomatic predementia stage that includes mild yet measurable impairment in memory and other cognitive abilities that exceeds normal aging (Alzheimer’s Association, 2020). Individuals with MCI are at higher risk for progression to dementia making early detection critical. The assessment and treatment of conditions like this, which disproportionately affect vulnerable populations, have been significantly hampered by the restrictions imposed by COVID-19, resulting in increased reliance on telehealth practices across disciplines. Clinical research operations have been similarly affected.

Cognitive evaluations for dementia have been uniquely impacted by COVID-19, as the risk of conducting traditional in-person evaluations might outweigh the benefits. Although the need for a neuropsychological evaluation may not generally be as urgent as other medical evaluations, it remains a critical component for differential diagnosis of dementia and to monitor disease progression (Schroeder et al., 2019).

Prior to COVID-19, the American Psychological Association (APA) and the Joint Task Force for the Development of Telepsychology Guidelines for Psychologists (2013) provided guidelines for remote services. However, due to the unprecedented nature of what the healthcare system is facing, such guidelines might not be fully applicable, leaving providers with uncertainty on how to address these pressing challenges. In response to the crisis, an advocacy team established by the Interorganizational Practice Committee (IOPC; Bilder et al., 2020) proposed a set of provisional guidelines as the framework for future formal teleneuropsychological guidelines. Teleneuropsychology was defined as the application of audiovisual technologies that allow for remote clinical encounters to conduct neuropsychological evaluations. The recommendations assisted with some of the challenges encountered by this modality.

In light of the ongoing crisis, the Centers for Medicare and Medicaid Services (CMS) have waived key telehealth requirements, allowing Medicare beneficiaries to receive services from home with fewer restrictions. CMS has also reimbursed telehealth services as if they were in-person visits (U.S. Congress, 2020). Patient privacy regulations have also been relaxed and the Health and Human Services (HHS) Office for Civil Rights has provided guidelines to temporarily permit the use of videoconferencing platforms such as FaceTime, Zoom, and Skype, which are not compliant with Health Insurance Portability and Accountability Act (HIPAA).

The purpose of this paper is to propose a novel Neuropsychological virtual protocol that is based on the existing literature but that also includes tailored recommendations to address the unique challenges faced during COVID-19. Our aim is to improve guidance on how to implement teleneuropsychology, by providing practical recommendations.

## What We Know About Teleneuropsychology

### Empirical Support for a Teleneuropsychology Protocol

Research examining the comparability of scores obtained *via* telehealth or in person has provided support for several standard neurocognitive instruments (Cullum et al., 2006; Barton et al., 2011; Parmanto et al., 2013; Smith et al., 2017; Wright, 2020). Verbal list learning tasks, such as the Hopkins Verbal Learning Test-Revised (Benedict et al., 1998), and auditory attention tasks, such as Digit Span (Wechsler, 2008), are well-suited for remote administration (Hildebrand et al., 2004). A meta-analysis and systematic review conducted by Brearly et al. (2017) of 497 participants across 12 studies provided support for the use of teleneuropsychology, particularly for verbal tasks, as the difference between in-person and telehealth administration had very modest effect sizes that were not statistically significant. This study supported the use of digit span, list learning, and verbal fluency tasks. With regards to dementia evaluations, various studies, including a rather large one conducted by Cullum et al. (2014), have supported the validity of teleneuropsychological evaluations in differentiating cognitively unimpaired individuals from those with varying levels of impairment (Wadsworth et al., 2018).

A recent systematic review conducted by Marra et al. (2020) provided strong support for the remote administration of cognitive screeners, such as the Mini Mental Status Examination (MMSE) and the Montreal Cognitive Assessment, for older adults 65 years of age and above (MoCA; Nasreddine et al., 2005) and moderate-to-strong evidence for the remote administration of all aspects of Digit Span (Wechsler, 2008), the HVLT-R (Benedict et al., 1998), the Clock Drawing Test, and the Boston Naming Test (Kaplan et al., 1983). In terms of language-based tasks, letter fluency demonstrated excellent validity, while category fluency had moderate support. Authors also pointed out that other tests, such as the Oral Trail Making Test – Parts A and B (Monsell et al., 2016), Brief Visuospatial Memory Test – Revised (Benedict et al., 1996), The Token Test (De Renzi and Vignolo, 1962), and Ponton-Satz Spanish Naming Test (Pontón et al., 1996), had promising results, but were evaluated by very few small studies.

### Limitations of Teleneuropsychology

The IOPC guidelines (Bilder et al., 2020) recognized that in-home assessment and the lack of a controlled environment would likely result in a novel challenge for neuropsychologists who would be providing these services. Importantly, the studies conducted about teleneuropsychology were done under very different circumstances that the ones society is facing today. As stated by the Joint Task Force for the Development of Telepsychology Guidelines for Psychologists (2013), “psychologists make every effort to ensure that ethical and professional standards of care and practice are met.” This means that despite the ongoing challenges and limited guidance, practitioners are required to balance the potential benefits of using teleneuropsychology (e.g., not delaying services, obtaining services from the comfort of their home, etc.) with its limitations/barriers (e.g., potential threats to validity, limitations in behavioral observations, etc.) on a case-by-case basis. Even more challenging, practitioners need to also consider alternate methods to evaluate individuals who have no access to home computer technology, since some types of data can be obtained through other methods, such as telephone assessments.

## University of Miami Proposed Teleneuropsychology Protocol

### Selecting a Telehealth Platform

Prior to initiating teleneuropsychology, the examiner must select a HIPAA-compliant videoconference platform that aligns with state regulations and IOPC guidelines (Bilder et al., 2020). Modification of the Informed Consent (IC), so it clearly states the limitations of the teleneuropsychological evaluation, and the modifications made to implement this modality are key prior to adopt services. As stated by the Joint Task Force for the Development of Telepsychology Guidelines for Psychologists (2013), “Psychologists strive to obtain and document informed consent that specifically addresses the unique concerns related to the telepsychology services they provide, including applicable laws and regulations, as well as organizational requirements pertinent to this area.” Grosch et al. (2011) recommended that the consent should contain several components including an explanation of the nature and purpose of the assessment, limits of confidentiality, and how those limits may apply to security of information transmitted over the internet or videoconferencing platform. Also, it is appropriate to include a statement regarding the modifications made to the standard administration. The neuropsychologist should go over the IC prior to the evaluation to address any questions regarding the validity of assessments.

### The Clinical Interview

We recommend using a desktop computer or a laptop built-in audio and camera systems with a screen monitor greater than or equal to 13 inches for the evaluation. Standard-size iPads are also acceptable, but are less preferred. To minimize distraction, the examiner should utilize a standard Zoom background, which mimics a standard office setting.

The clinical interview occurs remotely prior to the testing session and is conducted by the attending neuropsychologist and neuropsychology fellow *via* a secure Zoom platform. As with face-to-face intakes, we recommend having at least one family member or close friend present to corroborate and provide information about the examinee’s the history and functioning. This person can also assist with connectivity issues if needed. One of the advantages of the Zoom platform is that it allows for multiple users to join, even those who reside out of state. Upon obtaining consent from the patient or participant, the provider can invite other family members to the meeting by sharing the link. The clinical interview should determine whether the patient or participant is a good candidate for teleneuropsychology.

### Appropriateness for Teleneuropsychological Testing

Any teleneropsychological assessment must include an examination of the potential benefits and risks of providing such services with consideration of the patient’s particular needs, as well as ethical and multicultural factors (Cirone et al., 2010; Bilder et al., 2020). Our protocol includes initial assessment of an individual’s global level of cognition, clinical status, sensory barriers, and connectivity/devise accessibility prior to deciding if a particular patient or participant is an appropriate candidate for teleneuropsychology services. In addition to reviewing the referral question, the medical chart (if possible), and any other available data prior to the appointment, we also conduct a formal screening during the clinical interview. If the neuropsychologist believes that the patient or participant would benefit better from a face-to-face evaluation, our team documents this and recommends delaying the evaluation until feasible. Scott Kruse et al. (2018) identified technology issues and lack of computer literacy as the most important barriers for telehealth services. Based on our recent experience, we have found that providing a phone call prior to the initial evaluation with the purpose of helping them set up the virtual-platform, document which type of hardware the patient/participant has, and address any issues related to their device or connectivity prior to the day of the appointment has been useful in overcoming any barriers that can hinder the evaluation. This also provides information on their level of proficiency and help estimate how much time will be needed it to assist with connectivity and which battery should be used.

The evaluation should be conducted in a private environment, to reduce the possibility of breaches of privacy and confidentiality. We recommend that the neuropsychologist educates the examinee regarding the importance of being home for the evaluation and, if possible, accompanied by only one family member who can assist them (e.g., poor connectivity and technological problems) as needed. If the examinee resides with other family members, we encourage them to complete the evaluation in a private room. For our protocol, patients and participants will need the following materials for the assessment: four sheets of white printer paper, a pen, and pencil, a fully charged computer with camera and audio capabilities, and a table/desk. We ask examinees if they have these materials prior to the testing session, so we can mail them instruments ahead of time if needed.

### The University of Miami Teleneuropsychology Battery

Our battery includes measures that have been supported by previous research. See Table 1 for a list of tests utilized along with their corresponding domains. Due to the fatigue associated with video-conference meetings, our battery does not exceed 120 min. If the evaluation requires more than 120 min, the examinee is asked to join again on another day to complete the evaluation process. Our modified CNSA consent form is available through the Epic medical record system, which allows patients/participants to access and sign the consent form prior to meeting with the neuropsychologist. In addition, prior to commencing the session, the neuropsychologist goes over the consent form to explain the differences between the two modalities.

**Table 1:** Potential teleneuropsychological assessments.

Cognitive domain	Assessment
Global/Screener	Mini Mental State Examination (MMSE)
Global/Screener	Montreal Cognitive Assessment (MoCA, Audio-Visual Conference version)
Global/Screener	Addenbroke’s Cognitive Examination-III Remote Administration
Attention	Oral Trail Making Test – Parts A and B
Working Memory	Digit Span (Wechsler Adult Intelligence Scale – Fourth Edition; WAIS-IV)
Language	Boston Naming Test – Second Edition (BNT-2)
Language	Controlled Oral Word Association Test (COWAT)
Language	Animal Naming
Language	Vocabulary (WAIS-IV)
Premorbid Intelligence	Word Reading (Wide Range Achievement Test-4)
Learning and Memory	Hopkins Verbal Learning Test – Revised (HVLT-R)
Learning and Memory	California Verbal Learning Test – Third Edition (CVLT-3)
Learning and Memory	Logical Memory (Wechsler Memory Scale - Fourth Edition; WMS-IV)
Cognitive Stress Test	Loewenstein-Acevedo Scales for Semantic Interference and Learning (LASSI-L, Modified Version)
Cognitive Stress Test	Loewenstein-Acevedo Scales for Semantic Interference and Learning – Brief Computerized Version (LASSI-BC)
Executive Functioning	Clock Drawing
Abstract Reasoning	Similarities (WAIS-IV)
Emotional Functioning	Beck Depression Inventory – Second Edition (BDI-II)
Emotional Functioning	Beck Anxiety Inventory (BAI)
Emotional Functioning	Geriatric Depression Scale (GDS)
Emotional Functioning	Mini International Neuropsychiatric Interview (MINI, selected subscales)
Activities of Daily Living	Functional Assessment Questionnaire (FAQ)
Activities of Daily Living	Independent Living Scales (ILS, selected subtests)

In accordance with the APA guidelines stating that sending stimulus materials is not an appropriate solution to the current crisis, unless approved by the test publisher, we have been utilizing digital stimuli solely as permitted by the developers. For instance, certain test companies have been offering free access to digital protocols and stimuli during the pandemic. We have been displaying stimuli and instructions *via* PowerPoint presentations or PDF files when permitted and following administration guidelines. We recommend that examiners use printed test protocols ahead of time so they can score as they would during a regular face-to-face evaluation.

Prior to initiating testing, we recommend creating a plan for what to do if the examinee unexpectedly loses connection during the evaluation. Our plan to manage potential issues of dysconnectivity include collecting information ahead of time, so we can email them a Zoom link *via* a secure email address if needed to help them reconnect.

Prior to assessment, examiners should test the audio and camera features and practice sharing their screens. It is important to remind examinees to turn off or silence their cellphones and plug in their computer chargers if they are working on laptops. If applicable, examinees should maximize their screens, so they cannot see unrelated things on their desktop screens to avoid distractions.

The practitioner’s ethical responsibility to maintain psychometric standards and test integrity is the same regardless of whether she or he works in a conventional setting or remotely, *via* telemedicine (Naglieri et al., 2004; Lievens, 2006; Barak and Buchanan, 2011). Therefore, it is important to ensure that teleneuropsychological administration mimics traditional face-to-face administration as much as possible with appropriate modifications when required. Added/modified instructions are occasionally needed for remote administration of some measures. For example, if the battery includes visual memory tests with copy and delayed recall conditions, it is key to instruct examinees to rip up their drawings in between trials to prevent them from cuing themselves prior to the delayed recall condition. Additionally, following the delayed recall condition, the drawing should be disposed of, to maintain test security. To score figure drawings, we ask examinees to hold up their drawings while we take a screen shot of the entire paper. Capturing all four corners of the page is important, as it allows us to see where figures are placed on the page and how they are rotated. We recommend that the examiner names the file immediately to avoid confusing the different trials later during the scoring process. When administering verbal memory tests, it is important to remind examinees that writing is not allowed prior to the presentation of the stimuli. In our experience, examinees with memory impairment may require this reminder multiple times throughout the evaluation. When assessing for orientation, it is important to instruct examinees to “look directly into the camera” to reduce the chance that they will look at a calendar or other external cue.

If examinees require assistance connecting to the session or manipulating materials due to physical or cognitive impairments, include a caregiver or family member present during the evaluation. If so, it is crucial to educate caregivers/family members to remain quiet and refrain from providing cuing, or information during the evaluation. Anyone assisting with the evaluation should sit in the background or an adjacent room to reduce interfere with the testing.

### The Report

The American Telemedicine Association recommends that reports indicate that the data were obtained by video modality, a description of any technical difficulties experienced during the session, and an explanation of the extent to which the data may have been compromised (Yellowlees et al., 2010). The IOPC guidelines for COVID-19 also recommend that neuropsychologists include clear statements about the limitations of the teleneuropsychological assessment and the potential impact of these limitations on diagnostic conclusions/recommendations. We recommend to provide details regarding the testing environment, external distractions/interruptions encountered, and observations regarding the examinee’s workstation. For the summary and recommendations sections, providers should consider the American Psychological Association (2020) guidance for telehealth services during COVID-19, which state that no single test score should ever make a clinical decision for psychologists, even under the most optimal conditions. To increase confidence in data interpretation and decision making, consider widening confidence intervals and tapping into the same construct with multiple measures. The rationale and limitations of the evaluation should be considered when formulating diagnostic impressions. Additionally, it is often helpful to recommend repeating the evaluation at a later time on site to capture any missing information.

## Discussion and Future Directions

Our teleneuropsychology protocol includes tests that were previously validated for telehealth delivery as well as novel cognitive stress tests that uniquely measures proactive interference and the ability to recover from semantic interference [Loewenstein-Acevedo Scales for Semantic Interference (LASSI-L); Curiel et al., 2013]. Since the COVID-19 outbreak, our center has successfully completed 61 teleneuropsychological evaluations with older adults (70% English language and 30% Spanish language) using the outlined protocol. Feedback obtained during debriefing evidenced great acceptance from patients/participants and a uniformly positive satisfaction with our teleneuropsychological evaluation. Reported advantages include shorter evaluations, more flexibility with appointments, and less worry about virus exposure. Regarding feedback sessions, we have also received very positive feedback with good acceptance from families/patients. An advantage of conducting tele-feedbacks is having family members join the visit from the comfort of their homes, even if they reside at another state. An important limitation for our teleneuropsychology protocol is the lack of formal data analysis and comparison to in office visits. However, the presented protocol is intended to provide a framework for future research.

We strongly believe that telemedicine will continue to expand after the COVID-19 pandemic, eventually becoming a modal form of healthcare delivery in certain settings. There is evidence to support the use of this modality which could be of great advantage to older adults who lack transportation, are socially isolated, present with physical impediments, live a great distance from a tertiary medical care center, or are vulnerable to contracting infections in person. As such, it is critical to expand on existing research in order to further validate and refine neuropsychological assessment for teleneuropsychology. Application of insights from computer science and other fields can make virtual assessments as comparable to face-face encounters as possible. Future studies should include larger sample sizes, under-represented populations, and validate a broader range of assessment methods. While existing data appear promising, further studies evaluating the reliability and validity of teleneuropsychology vs. traditional evaluations are required. For now, we recommend that examiners continue to consider evolving research, and the examinee’s unique situation, when deciding whether teleneuropsychology should be the preferred method.

## Author Contributions

All authors listed have made a substantial, and intellectual contribution to the work, and approved it for publication.

### Conflict of Interest

DL and RC are co-developers of intellectual property mentioned in the manuscript.

The remaining authors declare that the research was conducted in the absence of any commercial or financial relationships that could be construed as a potential conflict of interest.
